# Severe Hyponatremic Encephalopathy Induced by Unsupervised “Therapeutic” 3,4-Methylenedioxymethamphetamine Use in a 55-Year-Old Woman: A Diagnostic Pitfall

**DOI:** 10.7759/cureus.108360

**Published:** 2026-05-06

**Authors:** Pierre-Henri Woitrin, Pascale Lievens

**Affiliations:** 1 Emergency Department, Centre Hospitalier Régional site Meuse, Namur, BEL

**Keywords:** 4-methylenedioxymethamphetamine (mdma), acute hyponatremia, diagnostic bias, emergency medicine, toxic encephalopathy, toxicology

## Abstract

While the clinical archetype of 3,4-methylenedioxymethamphetamine (MDMA) toxicity is traditionally associated with young individuals in nightlife environments, particularly in nightclubs and rave settings, its emerging role in therapeutic research may contribute to increasing unsupervised use. As MDMA gains mainstream attention for its potential mental health benefits, particularly for posttraumatic stress disorder, its use may involve a broader demographic, including older individuals in non-recreational contexts. This transition may create a diagnostic pitfall for emergency physicians, particularly through representativeness bias - the tendency to judge a clinical situation based on similarity to a stereotypical category without regard to underlying base rates. When severe hyponatremia occurs in an older patient during a mundane social setting, toxic etiologies may be overlooked, as the presentation does not match the expected profile of MDMA toxicity typically seen in younger individuals. We report a life-threatening case of MDMA-induced hyponatremic encephalopathy in a 55-year-old woman, occurring in a private domestic setting. The patient developed severe hyponatremia with neurological deterioration following self-directed "therapeutic" MDMA use. This case highlights the need to reconsider the demographic and contextual profile of synthetic drug toxicity in the era of emerging psychedelic-assisted therapy research and underscores the importance of maintaining a broad differential diagnosis for unexplained hyponatremia with altered mental status, regardless of patient age or social context.

## Introduction

3,4-methylenedioxymethamphetamine (MDMA), commonly known as "ecstasy," is a synthetic phenethylamine valued for its stimulant and empathogenic properties. While historically associated with young adults in high-energy recreational settings [1], its consumption is linked to severe metabolic complications, most notably acute hyponatremia [2]. Numerous cases of MDMA-associated hyponatremia have been reported in the literature, particularly in the context of excessive fluid intake and inappropriate antidiuretic hormone secretion [2]. This potentially fatal electrolyte disturbance can manifest rapidly, even following the ingestion of a single dose.

The pathophysiology of MDMA-induced hyponatremia has traditionally been categorized as a form of the syndrome of inappropriate antidiuresis (SIAD). However, recent clinical data suggest that a drug-induced increase in oxytocin, rather than vasopressin alone, may play a key role [3]. Due to its structural similarity to vasopressin, oxytocin at supraphysiological concentrations can exert antidiuretic effects via renal V2 receptors [4]. When exacerbated by compulsive fluid intake, this mechanism leads to profound dilutional hyponatremia and subsequent cerebral edema [2].

The demographic profile of MDMA users is currently shifting. The "psychedelic renaissance" and successful phase 3 trials for post-traumatic stress disorder (PTSD) have fueled a perception of MDMA as a therapeutic tool [5]. This has led to an emergence of unsupervised "self-medication" among older populations outside of traditional recreational contexts.

This shift presents a significant diagnostic pitfall for emergency physicians. The representativeness bias - a cognitive shortcut where clinicians judge a situation based on a stereotypical profile - may lead to the exclusion of toxic etiologies when severe hyponatremia occurs in an older patient in a domestic setting [6].

## Case presentation

Initial presentation and physical examination

A 55-year-old woman with no prior psychiatric history or known substance use disorder was admitted to the emergency department following a sudden loss of consciousness during a private dinner party. Upon arrival, the patient was in a deep coma with a Glasgow Coma Scale (GCS) score of 6 (E1V1M4). Initial vital signs were as follows: blood pressure 143/72 mmHg, heart rate 102 beats per minute, respiratory rate 15 breaths per minute, oxygen saturation 96% on room air, and body temperature 37.1°C. Physical examination revealed no focal neurological deficits, no lateralizing signs, and no evidence of fever or hemodynamic instability. Rapid sequence intubation was immediately performed to ensure airway protection.

Naloxone was not administered during the initial management, as there was no clinical suspicion of opioid intoxication, notably in the absence of miosis and respiratory depression.

Diagnostic investigations

**Figure 1:**
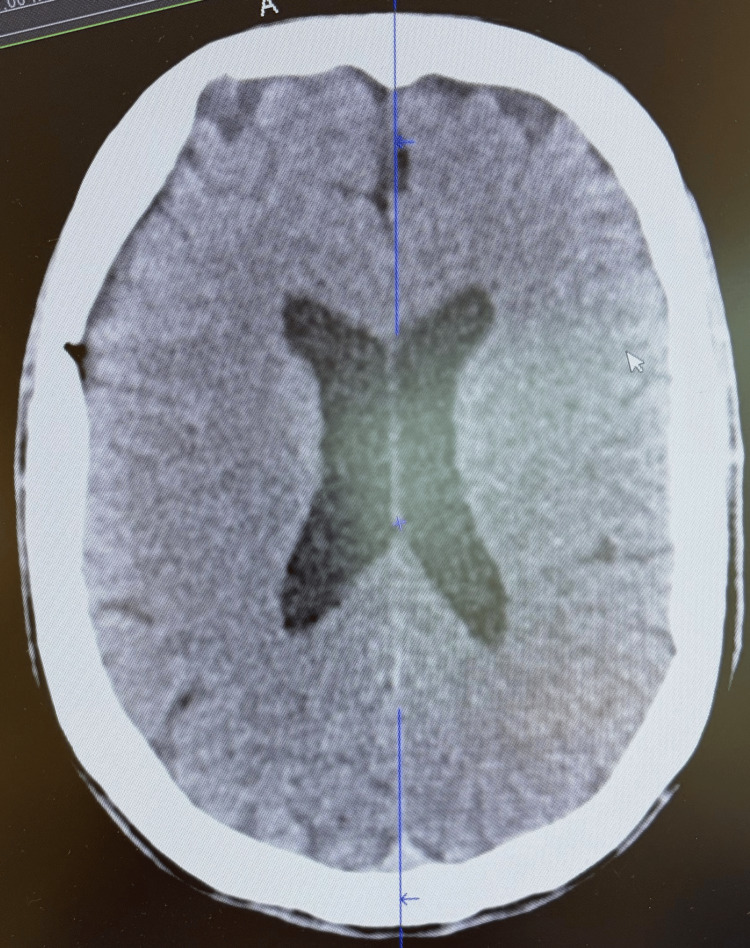
Non-contrast computed tomography (CT) of the head. Axial slice demonstrating no acute intracranial pathology, hemorrhage, or midline shift. The imaging was interpreted as normal, redirecting the diagnostic focus toward metabolic etiologies.

Upon admission, a non-contrast head CT scan was performed to rule out structural intracranial pathology; it showed no evidence of acute hemorrhage or mass effect (Figure 1). Initial laboratory investigations revealed a profound hyponatremia with a serum sodium of 122 mmol/L. Detailed metabolic and endocrine results, including the inappropriately high urine osmolality, are summarized in Table 1. Toxicology screening was performed via urinary immunoassay and subsequently confirmed by liquid chromatography-mass spectrometry, which was positive for MDMA (Table 1).

**Table 1:** Admission Laboratory Investigations. Summary of serum chemistry, endocrine profile, and toxicology results. Note the severe hyponatremia, high urine osmolality, and positive 3,4-methylenedioxymethamphetamine (MDMA) screening.

Laboratory Investigation	Result	Reference Range	Units
Serum Chemistry			
Sodium	122	135 - 145	mmol/L
Potassium	3.56	3.5 - 5.0	mmol/L
Chloride	86	98 - 108	mmol/L
Bicarbonate	16.7	22 - 29	mmol/L
Osmolality (Calculated)	254	275 - 295	mOsm/kg
Creatinine	0.73	0.5 - 0.9	mg/dL
C-Reactive Protein (CRP)	0.7	0.0 - 5.0	mg/L
Endocrine Profile			
Cortisol (Random)	50.5	6.0 - 18.0	µg/dL
Thyroid-stimulating hormone (TSH)	0.01	0.20 - 3.50	mU/L
Free T4	12.1	9.2 - 16.8	pg/mL
Urinalysis			
Urine Osmolality	571	50 - 1400	mOsm/kg
Urine Sodium	113	-	mmol/L
Toxicology Screening			
Urine Methamphetamine	Positive	Negative	-
Serum Ethanol	Negative	Negative	g/L
3,4-methylenedioxymethamphetamine (MDMA) (Confirmation)	Detected	Negative	-

Management and clinical course

Management focused on the neurological emergency. A 100 mL bolus of 3% hypertonic saline was administered, followed by a controlled infusion. Serum sodium levels were monitored every four hours; the concentration rose from 122 mmol/L at admission to 128 mmol/L within the first 24 hours. This correction rate of 6 mmol/L/day was strictly maintained to mitigate the risk of osmotic demyelination syndrome.

The patient’s neurological status improved rapidly, leading to successful extubation on Day 2. Upon recovery, the patient disclosed that she had ingested MDMA for unsupervised "therapeutic" purposes to manage chronic anxiety, based on information she had encountered online. This was her third experience with the substance. Witnesses reported excessive fluid intake during the evening.

A clearer chronological sequence of events was established, suggesting that MDMA ingestion followed by excessive fluid intake led to acute hyponatremia and subsequent neurological deterioration.

## Discussion

Altered mental status is a frequent and high-risk presentation in the emergency department, requiring rapid identification of life-threatening etiologies. Among metabolic causes, acute hyponatremia represents a medical emergency, particularly when associated with neurological symptoms such as seizures, coma, or signs of cerebral edema. Early recognition and prompt management are essential to prevent irreversible neurological damage.

In this case, the initial presentation was misleading. The patient was a 55-year-old woman with no reported history of substance use, presenting during a private dinner party - a context atypical for recreational MDMA exposure. This non-recreational setting likely contributed to a lower initial suspicion of toxicological causes. The absence of a typical "recreational" profile illustrates a common cognitive error in emergency medicine known as representativeness bias, in which clinicians rely on stereotypical patterns rather than considering the full spectrum of possible etiologies.

3,4-Methylenedioxymethamphetamine is a well-recognized cause of acute hyponatremia. The underlying mechanism has traditionally been attributed to inappropriate antidiuresis consistent with syndrome of inappropriate antidiuretic hormone secretion (SIADH), often exacerbated by excessive fluid intake [2]. Similar cases of MDMA-induced hyponatremia have been reported in the literature, often associated with excessive fluid intake and SIADH-like mechanisms [2].

However, recent evidence suggests a more complex mechanism. Emerging data indicate that MDMA-induced hyponatremia may be mediated, at least in part, by an increase in oxytocin levels rather than vasopressin alone [4]. Due to its structural similarity to vasopressin, oxytocin can exert potent antidiuretic effects via renal V2 receptors [3].

From a clinical standpoint, this distinction does not immediately alter acute management, which remains centered on controlled correction of serum sodium to prevent osmotic demyelination syndrome, in accordance with current guidelines [7,8]. Management typically involves cautious correction with hypertonic saline in severe cases, with close monitoring of serum sodium levels to avoid overly rapid correction and the risk of osmotic demyelination syndrome.

This emerging pattern represents a new challenge for emergency physicians. Patients may not spontaneously disclose substance use, particularly when perceived as therapeutic rather than recreational. As illustrated in this case, delayed disclosure can hinder timely diagnosis and contribute to severe clinical presentations. Systematic toxicology screening should therefore be considered in any patient presenting with unexplained hyponatremia and altered mental status, regardless of age, social context, or initial history [6].

This case serves as a reminder that the demographic and contextual profile of synthetic drug toxicity is evolving, and clinical vigilance must adapt accordingly

## Conclusions

This case highlights an atypical presentation of MDMA toxicity in an older patient in a non-recreational setting. It underscores the risk of diagnostic bias and the importance of maintaining a high index of suspicion for toxicological causes in patients presenting with unexplained hyponatremia and altered mental status. The growing trend of unsupervised “therapeutic” use may represent an emerging source of severe presentations in the emergency department.
